# DNA oxidation after exercise: a systematic review and meta-analysis

**DOI:** 10.3389/fphys.2023.1275867

**Published:** 2023-10-31

**Authors:** Mengxin Ye, Luthfia Dewi, Yu-Chieh Liao, Andrew Nicholls, Chih-Yang Huang, Chia-Hua Kuo

**Affiliations:** ^1^ College of Physical Education and Science, Zhejiang Normal University, Jinhua, China; ^2^ Laboratory of Exercise Biochemistry, University of Taipei, Taipei, Taiwan; ^3^ Cardiovascular and Mitochondria Related Disease Research Center, Hualien Tzu Chi Hospital, Buddhist Tzu Chi Medical Foundation, Hualien, Taiwan; ^4^ Department of Medical Research, China Medical University Hospital, China Medical University, Taichung, Taiwan; ^5^ Department of Biotechnology, Asia University, Taichung, Taiwan; ^6^ Center of General Education, Buddhist Tzu Chi Medical Foundation, Tzu Chi University of Science and Technology, Hualien, Taiwan; ^7^ Graduate Institute of Basic Medical Science, China Medical University, Taichung, Taiwan; ^8^ School of Physical Education and Sports Science, Soochow University, Suzhou, China

**Keywords:** oxidative DNA damage, 8-OHdG, 8-oxodG, free radicals, ROS, 8-Hydroxy-2’-deoxyguanosine, 8-oxo-7, 8-dihydro-2’-deoxyguanosine

## Abstract

**Purpose:** 8-Hydroxy-2′-deoxyguanosine (8-OHdG) is a byproduct of DNA oxidation resulting from free radical attacks. Paradoxically, treatment with 8-OHdG accelerates tissue healing. The aim of this study is to quantify the 8-OHdG response after a single session of exercise in both trained and untrained adults.

**Methods:** A systematic review and meta-analysis of exercise intervention studies measuring changes in blood 8-OHdG following resistance exercise and aerobic exercise were conducted. The literature search included Web of Science, PubMed, BASE, and Scopus, with publications up to February 2023 included. Subgroup analysis of training status was also conducted.

**Results:** Sixteen studies involving 431 participants met the eligibility criteria. Resistance exercise showed a medium effect on increasing circulating 8-OHdG levels (SMD = 0.66, *p* < 0.001), which was similar for both trained and untrained participants. However, studies on aerobic exercise presented mixed results. For trained participants, a small effect of aerobic exercise on increasing circulating 8-OHdG levels was observed (SMD = 0.42; *p* < 0.001). In contrast, for untrained participants, a large effect of decreasing circulating 8-OHdG levels was observed, mostly after long-duration aerobic exercise (SMD = −1.16; *p* < 0.05). Similar to resistance exercise, high-intensity aerobic exercise (5–45 min, ≥75% VO_2max_) significantly increased circulating 8-OHdG levels, primarily in trained participants.

**Conclusion:** Pooled results from the studies confirm an increase in circulating 8-OHdG levels after resistance exercise. However, further studies are needed to fully confirm the circulating 8-OHdG response to aerobic exercise. Increases in 8-OHdG after high-intensity aerobic exercise are observed only in trained individuals, implicating its role in training adaptation.

**Systematic Review Registration**: [https://Systematicreview.gov/], identifier [CRD42022324180]

## Introduction

An intense workout inevitably induces cellular damage and results in a transient increase in reactive oxygen species (ROS) (Balaban et al., 2005). However, the health benefits of exercise training are well documented in the scientific literature. This seeming contradiction suggests that an inherent mechanism must exist to gauge oxidative damage and prompt tissue repair to enhance fitness. Currently, ROS are considered essential signaling molecules for skeletal muscle adaptations to exercise challenges (Powers et al., 2010). High ROS levels are required for the mobilization of bone marrow stem cells, while low ROS levels promote the proliferation of quiescent stem cells, highlighting the physiological importance of ROS oscillation (Ludin et al., 2014). NADPH oxidases (Ferreira and Laitano, 2016), xanthine oxidases (Gomez-Cabrera et al., 2010), and mitochondria (Bejma and Ji, 1999) are the sources of ROS produced during and after exercise. Redox signaling that regulates exercise responses and adaptations predominantly occurs through tightly controlled signaling cascades (Radak et al., 2017; Henriquez-Olguin et al., 2020).

Transient ROS production induces a protracted elevation in 8-hydroxy-2′-deoxyguanosine (8-OHdG) as a byproduct of DNA oxidation (Valavanidis et al., 2009). Interestingly, treatment with 8-OHdG has been shown to speed up the healing process and increase the resolution of inflammation *in vivo* (Hyun et al., 2006; Takemura et al., 2010; Ock et al., 2011a; Ock et al., 2011b; Kim et al., 2011; Kim et al., 2021). This suggests that 8-OHdG may serve as a danger-associated molecular pattern (DAMP), triggering an immune response to promote tissue regeneration (Tamai et al., 2011; Vénéreau et al., 2015). Therefore, it is likely that 8-OHdG acts as a mediator for training adaptation, reducing recovery time following the next bout of exercise.

A previous systematic review and meta-analysis by Tryfidou et al. (2020) reported the acute effect of aerobic exercise and described the role of DNA oxidation on training adaptation. However, the question regarding whether the aerobic exercise response on 8-OHdG is different between trained and untrained individuals was not addressed in the study. Furthermore, no systematic review or meta-analysis reporting the effect of resistance exercise on circulating 8-OHdG levels is currently available. Resistance exercise is generally known to induce more muscle damage yet produces more muscle hypertrophy than aerobic exercise. Therefore, this study aims to investigate the impact of acute bouts of both types of exercise on circulating 8-OHdG levels, with a focus on the training status.

## Methods

This study was performed in accordance with the Preferred Reporting Items for Systematic Reviews and Meta-Analyses (PRISMA) guidelines (Moher et al., 2009) and was registered *a priori* in the PROSPERO database (CRD42022324180).

### Study selection and search strategy

We searched four databases (Web of Science, PubMed, BASE, and Scopus) for studies on the acute effects of exercise on 8-OHdG. All published studies were checked for the following criteria: full reports published in peer-reviewed journals, human intervention studies with estimated exercise intensity and duration, and articles containing keywords referring to the following terms (used in all possible combinations): “exercise,” “exercise training,” “acute exercise,” “physical activity,” “8-hydroxy-2-deoxyguanosine,” “8-oxo-2-deoxyguanosine,” “8-OHdG,” “8OHdG,” “8-OHG,” “8-oxo-dG,” “DNA damage,” “oxidative damage,” and “oxidative stress.” In the study, literature reporting non-specific oxidative damage markers irrelevant to DNA oxidation, such as protein carbonyl and F2-isoprostane, was excluded. Additionally, the reference lists of the included studies were reviewed, and a detailed search was carried out to identify all relevant studies, including all available publications up to February 2023. One investigator initially reviewed records generated from all databases and applied the inclusion/exclusion criteria to identify eligible studies; these were then agreed with at least three of the authors. Studies were included according to the PICOS model (Supplemental Table S1) (Brown et al., 2006). Acute exercise was defined as aerobic exercise or resistance exercise performed in a single session including short and sustained working capacity. For quantitative analysis, only studies utilizing blood samples were included. We excluded studies that lacked reporting on exercise intensity and duration, omitted follow-up time post-exercise, involved unhealthy participants (i.e., patients), or utilized animals for measurements. Studies that measured human skeletal muscle and those involving anaerobic exercise (including the Wingate test and sprinting) were discussed in the systematic review.

### Data extraction and quality assessment

Once the included studies were finalized, the data were categorized by the characteristics of participants (sample size and training status) and the exercise type (resistance and aerobic). Eligible studies were classified as aerobic exercise (intensity indicated by % VO_2max_) and resistance exercise (intensity indicated by % maximal muscle strength) according to the muscle contraction mode. Outcome values (8-OHdG) are expressed as % pre-exercise baseline as control. Duration >30 min is considered prolonged aerobic exercise. Assayed biomarkers and methods of DNA quantification were extracted. Due to variations in the analytical approach, 8-OHdG values by high-performance liquid chromatography (HPLC) and enzyme-linked immunosorbent assay (ELISA) were expressed as 8-OH-dG/10^6^ Gua or ng/mL. The records were imported into EndNote (version 20.1, Clarivate Analytic, Philadelphia, PA, United States) and were automated and manually screened. The effect size of the outcome after exercise was expressed as a standardized mean difference (SMD). If the mean values in the results of the full-text article were only presented in a figure format, we used WebPlotDigitizer (V.4.2. Texas, United States) to extract the data from the studies. The research divided the training status of the participants: those with long-term training experience (such as majored in physical education or long-term training in the gym) were classified as trained. Untrained participants were defined by the articles, and those who exercise less than three times a week and who are sedentary were undefined.

The quality of the included studies was assessed using five domains according to the revised Cochrane Risk of Bias tool for randomized trials consisting of 1) the randomization process, 2) deviations from intended interventions, 3) missing outcome data, 4) measurement of the outcome, and 5) selection of the reported result. The overall risk of bias was defined as “low risk” if all domains were at low risk of bias, “some concerns” if containing at least one domain at some concerns status, but not at high risk of bias for any domain, and “high risk” if at least one study was judged in some concerns for multiple domains.

### Data synthesis and analysis

The primary outcome was blood 8-OHdG induced by an acute bout of exercise. A detailed analysis was conducted by subgroup analysis to compare the differences against pre-exercise baseline in the 1) type of exercise (resistance and aerobic), 2) training status, 3) duration of aerobic exercise, and 4) intensity of aerobic exercise. Due to the limited number of literature works available, 8-OHdG in muscle samples was included only in the systematic review (Radák et al., 1999; Fogarty et al., 2013; Yasuda et al., 2015).

The mean, standard deviation (SD), and sample size of the group that only received exercise intervention were pooled. Forest plots were produced to display the effect size using SMDs and the overall effect of Z-score. If the literature reported the standard error (SE) only, SD was calculated using the following formula, where n represents the number of participants:

$$\text{SD}=\text{SE }x\;\sqrt{n}.$$

We assumed the heterogeneity of the data among studies, and therefore, a random-effects model was performed. We calculated the SMD according to Hedges’ adjusted g, as follows:

$$\text{SMD}=\frac{{\text{Mean}}_{\text{post}-}{\text{Mean}}_{\text{baseline}}}{{\text{SD}}_{\text{pooled}}}\left(1-\frac{3}{4N-9}\right),$$

where N represents the total sample size of post-intervention and baseline. The effect size was categorized into small (SMD = 0.2—0.5), medium (SMD = 0.5—0.8), and large (SMD >0.8) (Fritz et al., 2012; Hoaglin, 2016; Lamberink et al., 2018), and pooled SD was calculated by

$$\text{SDp}=\sqrt{{\left({\text{SD}}_{\text{baseline}}\right)}^{2}+{\left({\text{SD}}_{\text{post}}\right)}^{2}-2\;x\;r\;x\;{\text{SD}}_{\text{baseline}}\;x\;{\text{SD}}_{\text{post}},}$$

where r represents the correlation coefficient. The 95% confidence interval including “0” referred to non-statistical significance (Faraone, 2008). An increasing 8-OHdG in response to exercise was indicated by a positive SMD. To the contrary, a negative SMD represented a decreasing effect of exercise. The overall effect size using the Z-score was considered significant at *p* < 0.05 (Lee, 2016). Tau-squared (τ^2^), chi-squared Cochran’s Q (𝒳^2^) test, and I^2^ statistic indicated the statistical heterogeneity. The value of τ^2^ > 1 demonstrated the variability between studies. The Q test measured the variation around a weighted mean, in which *p*-value <0.10 was considered to be indicative of significant heterogeneity. I^2^ percentage reflected the consistency of results across the studies. I^2^ was classified into no important heterogeneity (I^2^ = 0–30%), moderate heterogeneity (I^2^ = 31–49%), substantial heterogeneity (I^2^ = 50–74%), and considerable heterogeneity (I^2^ = 75–100%) (Higgins et al., 2003). The analysis was performed using Review Manager (RevMan version 5.4.1, Cochrane: United Kingdom).

## Results

### Selection process

**FIGURE 1:**
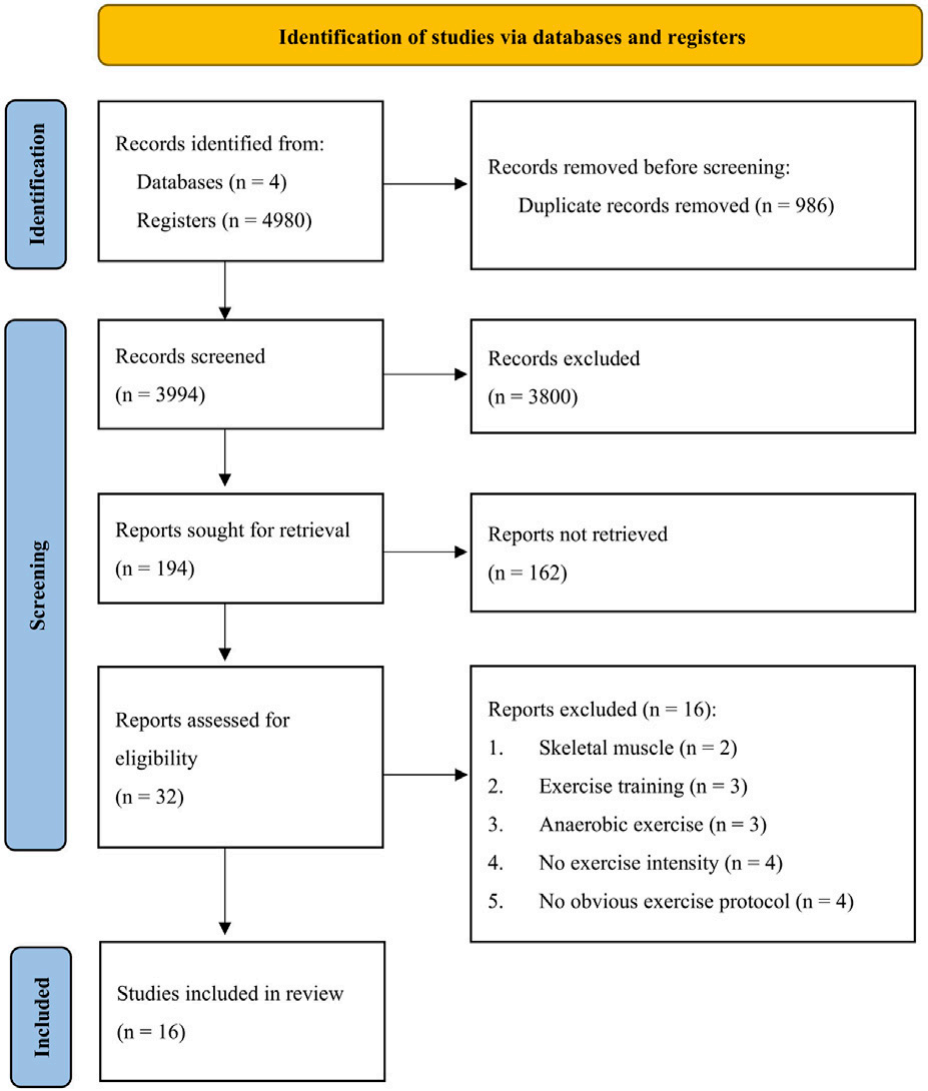
PRISMA flow chart.

The number of identified articles and the selection process from the four databases are shown in Figure 1. We retrieved 986 duplicates from 4,980 published articles in the database search and excluded 3,800 records after title screening, leaving 194 records for abstract screening. For quantitative analysis, we removed 162 records after abstract screening according to the exclusion criteria, resulting in 32 full-text articles for eligibility assessment. A total of 16 studies (431 participants) were finally included for the meta-analysis after exclusion of three long-term training studies (Margaritis et al., 2003; Mrakic-Sposta et al., 2015; Atli, 2021), three studies involved with Wingate tests (Cuevas et al., 2005; Shi et al., 2007; Serkan, 2011), four studies not reporting exercise intensity (Inoue et al., 1993; Asami et al., 1998; Hamurcu et al., 2010; Meihua et al., 2018), and four studies not reporting the exercise protocol (Giacomo et al., 2009; Mergener et al., 2009; Aires et al., 2010; Neubauer et al., 2010). Studies reporting the levels of 8-OHdG in human skeletal muscle were used for the systematic review (Radák et al., 1999; Yasuda et al., 2015).

### Quality assessment in individual studies

Among the included eligible studies, two studies scored in the moderate-risk bias (Saritaş et al., 2011; Sarmiento et al., 2016) and 14 studies scored in the low-risk bias (Supplementary Table S2) (Sacheck et al., 2003; Sato et al., 2003; Bloomer et al., 2005; Bloomer et al., 2006; Itoh et al., 2006; Bloomer et al., 2007; Shi et al., 2007; Harms-Ringdahl et al., 2012; Fogarty et al., 2013; Pittaluga et al., 2013; Ra et al., 2013; Çakır-Atabek et al., 2015; Arazi et al., 2019; Mohammadjafari et al., 2019).

### Study characteristics

Supplementary Table S3 summarizes participant characteristics including sample sizes, training status, exercise types (intensity and duration), specimens, post-exercise follow-up time, and percent changes in 8-OHdG levels with 431 blood samples. The average age of participants of eligible studies ranged from 21 to 71 years. Nine studies recruited trained participants (Bloomer et al., 2005; Bloomer et al., 2006; Bloomer et al., 2007; Shi et al., 2007; Saritaş et al., 2011; Pittaluga et al., 2013; Sarmiento et al., 2016; Arazi et al., 2019; Mohammadjafari et al., 2019). Five studies recruited untrained participants (Sacheck et al., 2003; Itoh et al., 2006; Harms-Ringdahl et al., 2012; Fogarty et al., 2013; Ra et al., 2013). Two studies included both untrained and trained participants (Sato et al., 2003; Çakır-Atabek et al., 2015).

### Exercise type

Intensity for aerobic exercise (nine studies, 230 participants) ranged from 50% to 100% VO_2max_. Intensity for resistance training (eight studies, 201 participants) ranged from 50%–100% of 1RM (one-repetition maximum). One study conducted both types of exercise (Bloomer et al., 2005). Three studies measured 8-OHdG in human skeletal muscle: one study used aerobic exercise (50% VO_2max,_ 5 h) (Yasuda et al., 2015), and two studies used resistance exercise (60% 1RM and 100-repetition maximal knee extension) as challenges (Radák et al., 1999; Fogarty et al., 2013). Three articles reporting the Wingate test as an exercise challenge were excluded from aerobic exercise in the meta-analysis but included in the systematic review (Cuevas et al., 2005; Shi et al., 2007; Serkan, 2011).

### Effect of resistance exercise on blood 8-OHdG levels

**FIGURE 2:**
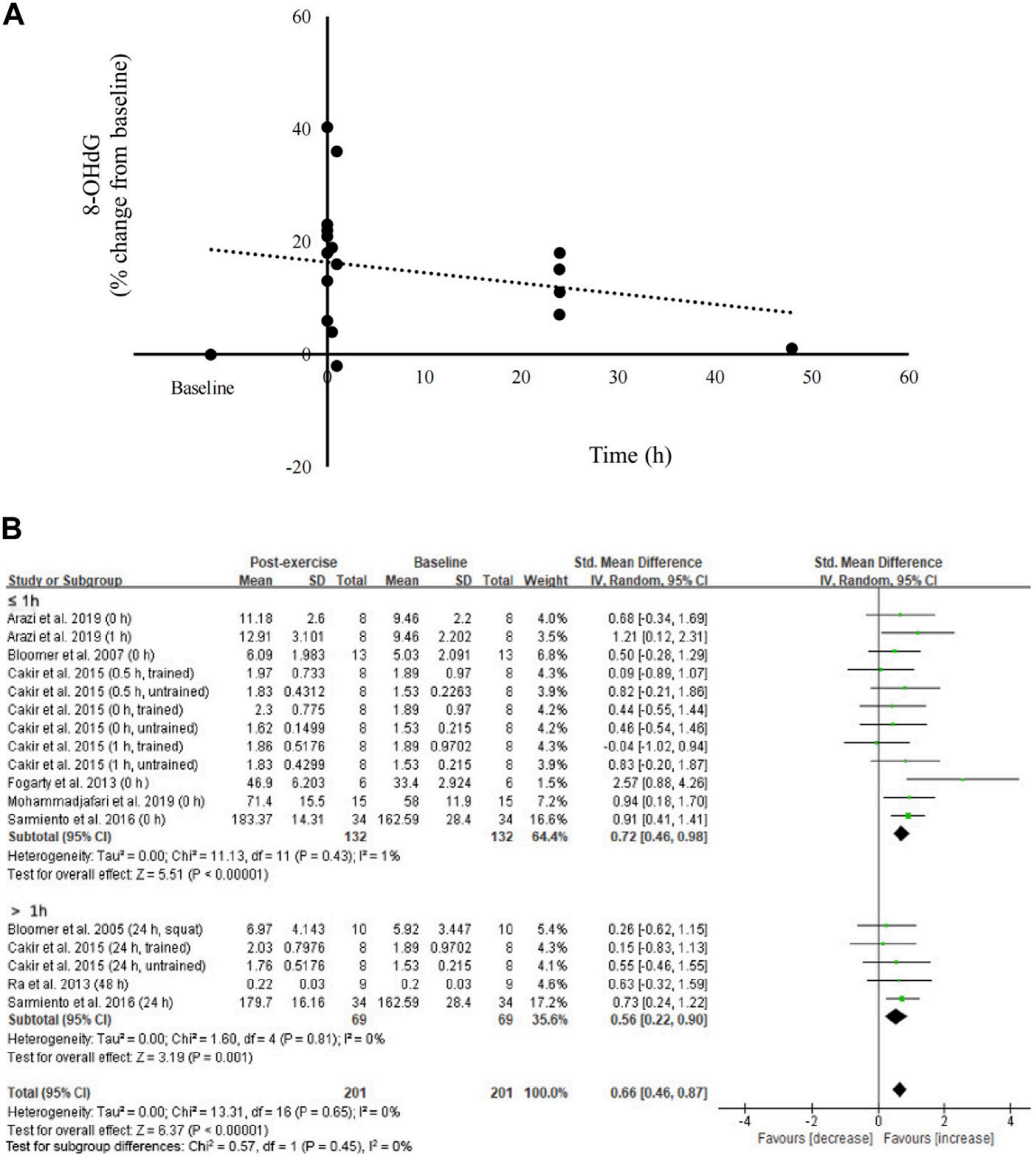
Effects of resistance exercise on circulating 8-OHdG. 8-OHdG levels increased immediately after resistance exercise and diminished thereafter **(A)**. Medium effect of resistance exercise on 8-OHdG **(B)**. 8-OHdG, 8-hydroxy-2′-deoxyguanosine.

The percent changes in circulating 8-OHdG levels from pre-exercise baseline up to 48 h from eight studies (Bloomer et al., 2005; Bloomer et al., 2007; Fogarty et al., 2013; Ra et al., 2013; Çakır-Atabek et al., 2015; Sarmiento et al., 2016; Arazi et al., 2019; Mohammadjafari et al., 2019) are shown in Figure 2. The scatter plot indicates prominent increases in circulating 8-OHdG levels shortly after resistance exercise and seems to reverse thereafter in 48 h (n = 201) (Figure 2A). Subgroup analysis further indicates medium effects of resistance exercise on increasing circulating 8-OHdG levels ≤1 h after resistance exercise (Bloomer et al., 2007; Fogarty et al., 2013; Çakır-Atabek et al., 2015; Sarmiento et al., 2016; Arazi et al., 2019; Mohammadjafari et al., 2019) (SMD = 0.72; 95% CI: 0.46 to 0.98; *p* < 0.001) and >1 h after resistance exercise (Bloomer et al., 2005; Ra et al., 2013; Çakır-Atabek et al., 2015; Sarmiento et al., 2016) (SMD = 0.56; 95% CI: 0.22 to 0.90; *p* < 0.05) (Figure 2B).

### Effect of aerobic exercise on blood 8-OHdG levels

**FIGURE 3:**
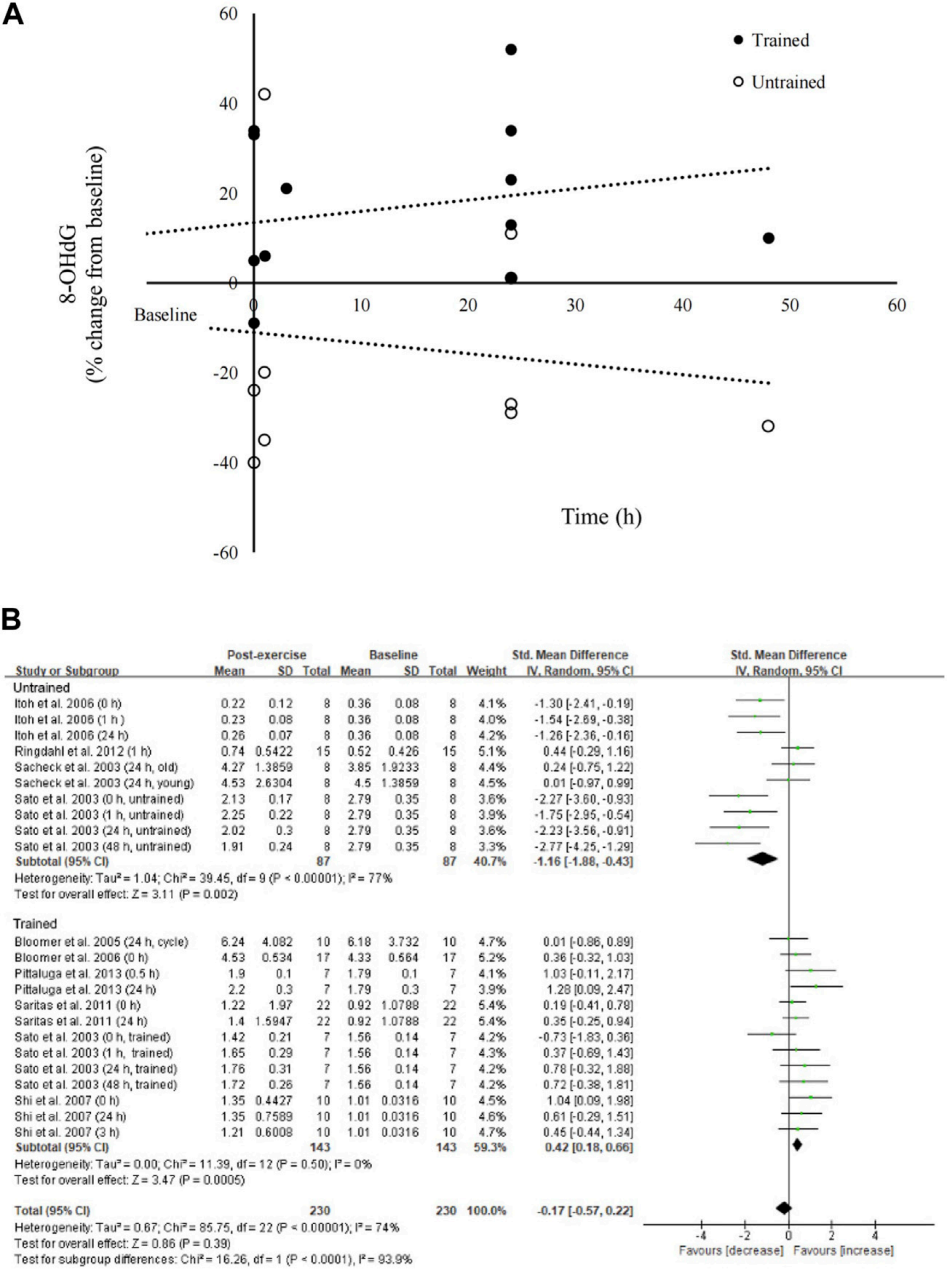
Effects of aerobic exercise on circulating 8-OHdG. Post-exercise changes in 8-OHdG are divergent for trained and untrained participants **(A)**. Subgroup analysis for trained and untrained participants further showed opposing response in 8-OHdG, resulting in no overall effect of aerobic exercise **(B)**. 8-OHdG, 8-hydroxy-2′-deoxyguanosine.

The effect of aerobic exercise on circulating 8-OHdG levels is shown in Figure 3. We observed an opposing response to aerobic exercise on circulating 8-OHdG levels for trained and untrained participants (230 samples). Subgroup analysis indicated a large effect of aerobic exercise on decreasing 8-OHdG levels in untrained participants (Sacheck et al., 2003; Sato et al., 2003; Itoh et al., 2006; Harms-Ringdahl et al., 2012) (SMD = −1.16; 95% CI: −1.88 to −0.43; *p* < 0.01). On the contrary, a small effect of aerobic exercise on increasing 8-OHdG levels for studies using trained participants was observed (Sato et al., 2003; Bloomer et al., 2005; Bloomer et al., 2006; Shi et al., 2007; Saritaş et al., 2011; Pittaluga et al., 2013) (SMD = 0.42; 95% CI: 0.18 to 0.66; *p* < 0.01).

### Duration and intensity effect of aerobic exercise

**FIGURE 4:**
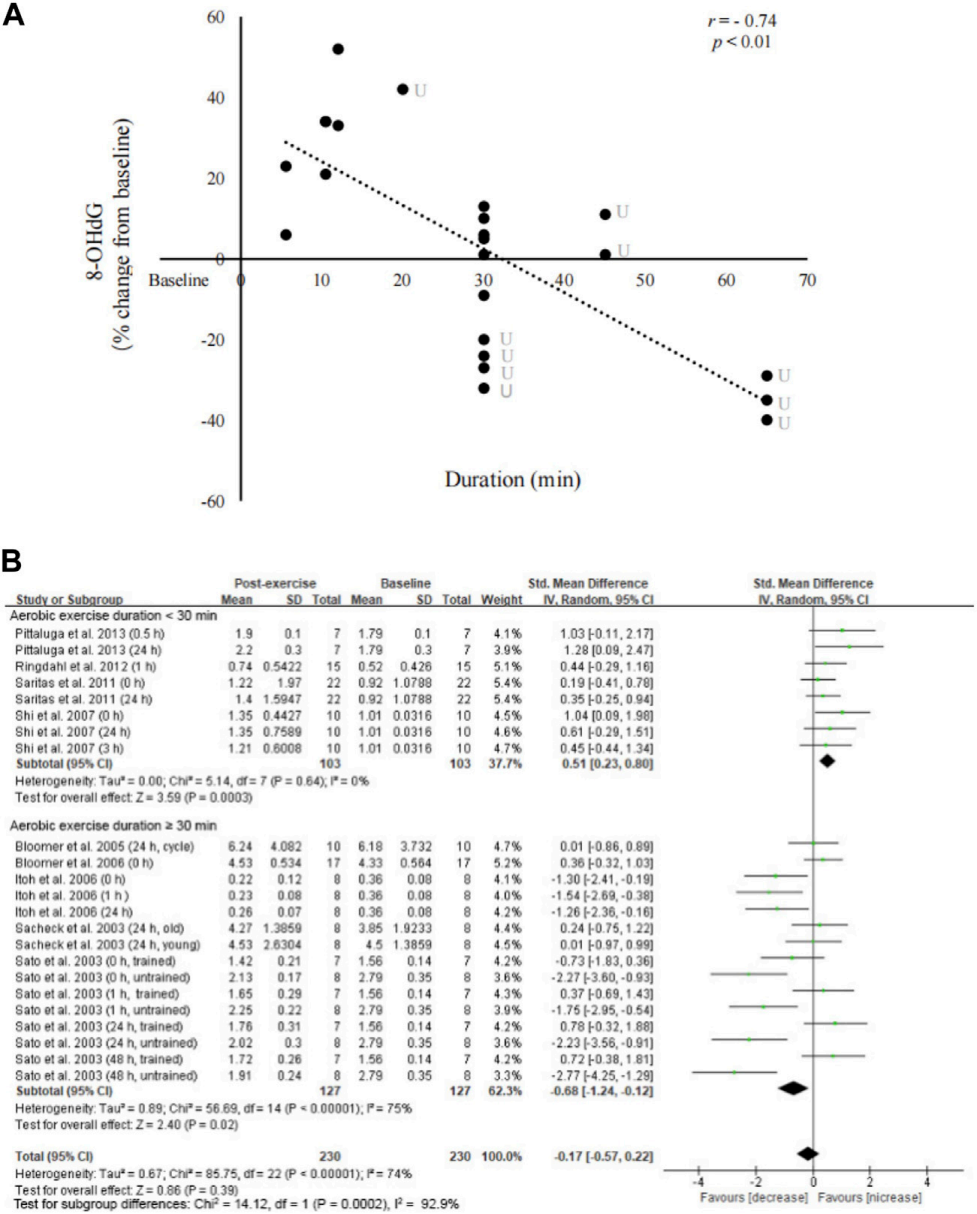
Linear relationship between exercise duration and circulating 8-OHdG. An inverse relationship between 8-OHdG and exercise duration was observed without considering exercise intensity and training status (*r* = −0.74, *p* < 0.001) **(A)**. Forest plots show significant increases in 8-OHdG after short-duration exercise (<30 min) and decreases in 8-OHdG after long-duration exercise (>30 min) **(B)**. 8-OHdG, 8-hydroxy-2′-deoxyguanosine; U, untrained.

There was a significant inverse relationship (*r* = −0.74, *p* < 0.01) between exercise duration and changes in circulating 8-OHdG levels from pre-exercise baseline (Figure 4). Subgroup analysis indicates a moderate effect of aerobic exercise on increasing circulating 8-OHdG levels when the exercise duration was <30 min (Shi et al., 2007; Saritaş et al., 2011; Harms-Ringdahl et al., 2012; Pittaluga et al., 2013) (SMD = 0.51; 95% CI: 0.23 to 0.80; *p* < 0.01). However, a moderate effect of aerobic exercise on decreasing circulating 8-OHdG levels was found when the exercise duration was >30 min (SMD = −0.68; 95% CI: −1.24 to −0.12; *p* < 0.05) (Sacheck et al., 2003; Sato et al., 2003; Bloomer et al., 2005; Bloomer et al., 2006; Itoh et al., 2006).

**FIGURE 5:**
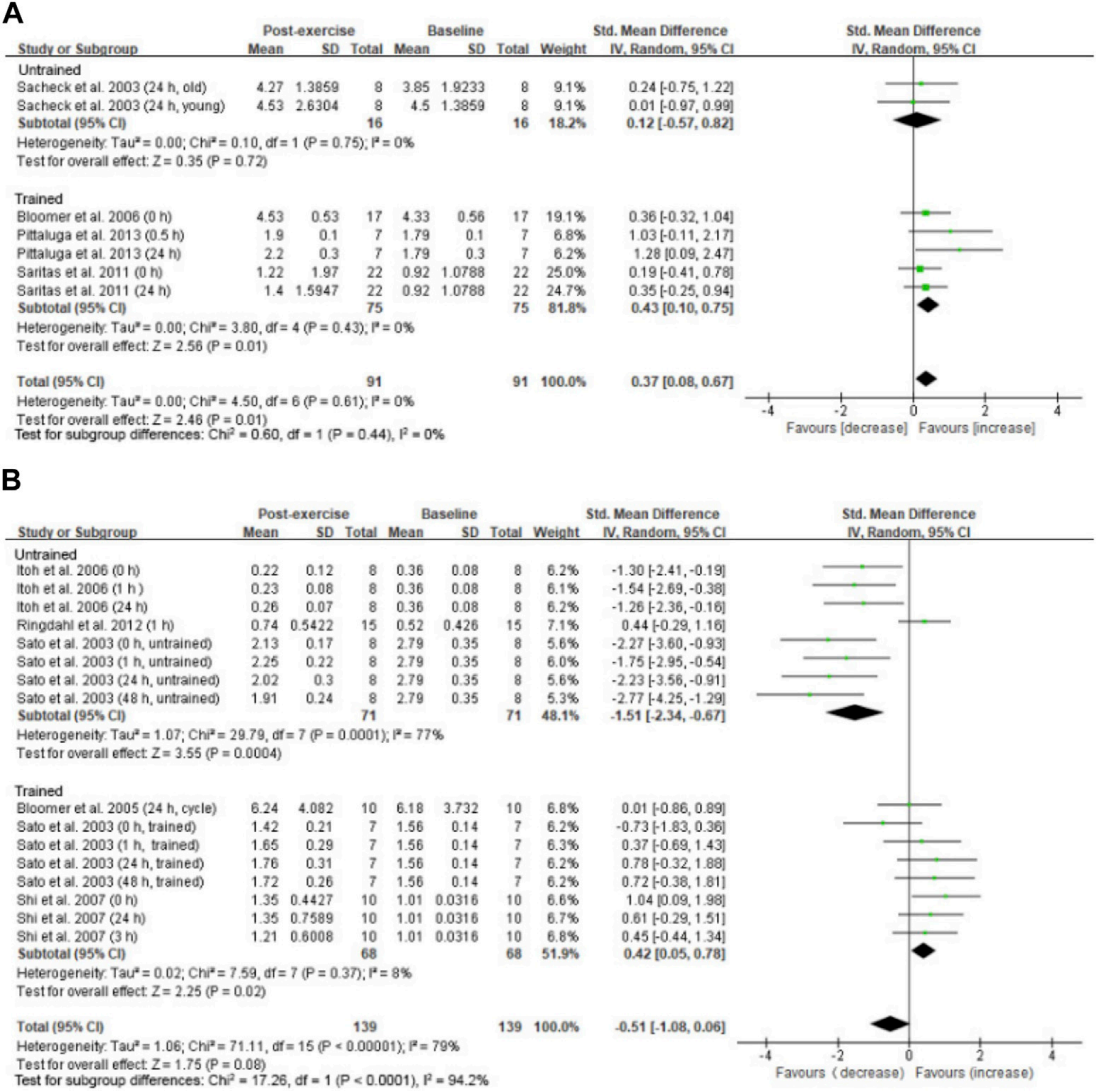
Intensity effect on circulating 8-OHdG after aerobic exercise. A small effect of increasing 8-OHdG was observed for trained participants performing high-intensity exercise (≥75% VO_2max_) regardless of the training status **(A)**. A large effect of decreasing 8-OHdG was observed for untrained participants performing low-intensity exercise (<75% VO_2max_) **(B)**. 8-OHdG, 8-hydroxy-2′-deoxyguanosine.

A small overall effect of high-intensity exercise (≥75%VO_2max_) on increasing 8-OHdG levels was observed (SMD = 0.37; 95% CI: 0.08 to 0.67; *p* = 0.01) (Sacheck et al., 2003; Bloomer et al., 2006; Saritaş et al., 2011; Pittaluga et al., 2013) (Figure 5A). This effect is contributed mostly from studies using trained participants (SMD = 0.43; 95% CI: 0.10 to 0.75; *p* = 0.01) (Bloomer et al., 2006; Saritaş et al., 2011; Pittaluga et al., 2013). Only one study involving high-intensity aerobic exercise used untrained participants (Sacheck et al., 2003). However, we observed a significant large effect of low-intensity aerobic exercise (<75% VO_2max_) on decreasing 8-OHdG levels from studies using untrained participants (SMD = −1.51; 95% CI: −2.34 to −0.67; *p* < 0.001) (Figure 5B) (Sato et al., 2003; Itoh et al., 2006; Harms-Ringdahl et al., 2012), whereas a small effect on increasing 8-OHdG levels was observed from the low-intensity studies using trained participants (SMD = 0.42; 95% CI: 0.05 to 0.78; *p* < 0.05) (Sato et al., 2003; Bloomer et al., 2005; Shi et al., 2007).

## Discussion

This is the first meta-analysis quantitatively assessing post-exercise DNA oxidation (based on 8-OHdG outcomes) comprising studies of both resistance exercise and aerobic exercise. The main findings of the study are as follows: 1) resistance exercise increases circulating 8-OHdG levels and diminishes in 48 h during recovery; 2) circulating 8-OHdG response to aerobic exercise varies depending on the training status of the participants. Despite significant increases in circulating 8-OHdG, resistance exercise and high-intensity aerobic exercise are generally known to provide greater muscle adaptation and survival benefits than low-intensity exercise (Byberg et al., 2009; Powers et al., 2020). Taken together with the observed anti-inflammatory action of 8-OHdG (Choi et al., 2007), the results of this study implicate the involvement of 8-OHdG molecules in training adaptation.

The levels of ROS response against exercise are apparently correlated with exercise volume and intensity. Circulating 8-OHdG level is determined by the dynamical balance between DNA damage and the repair mechanism. In human skeletal muscle, resistance exercise is able to elevate 8-OHdG levels up to 91% immediately after completion (Fogarty et al., 2013). It is generally known that resistance exercise induces significant muscle injury leading to neutrophil infiltration to initiate phagocytosis for debris clearance. Phagocytosis is the main source of ROS required for the lysis of damaged cells (Al-Shehri, 2021). Therefore, increases in circulating 8-OHdG levels could reflect the short burst of phagocytosis (high ROS) before entering the cell regenerative phase (low ROS) of the inflammation process. However, 8-OHdG inhibits inflammation. 8-OHdG is a competitive inhibitor that can bind to the GTP-binding site of Rac1 protein due to its structure similarity to GTP. Exogenous 8-OHdG treatment decreases Rac1-GTP and, therefore, inhibits Rac1 and lowers ROS (Ock et al., 2011a). During inflammation, Rac1 is an activator of NADPH oxidase for phagocytosis (Hordijk, 2006) and the STAT3/NF-κB signaling pathway (Myant et al., 2013). Therefore, dynamical changes in 8-OHdG may involve with time required to resolve inflammation.

An intriguing finding of the study is the opposing response in post-exercise 8-OHdG between trained (Sato et al., 2003; Bloomer et al., 2006; Shi et al., 2007; Saritaş et al., 2011; Pittaluga et al., 2013) and untrained individuals (Sacheck et al., 2003; Sato et al., 2003; Itoh et al., 2006; Harms-Ringdahl et al., 2012) following aerobic exercise. The underlying mechanism to explain this discrepancy may involve their differences in muscle mitochondria density. Mitochondrial DNA content and NADH oxidase are significantly greater in aerobically trained muscles than those in untrained muscles (Menshikova et al., 2006; Granata et al., 2018). Mitochondrial DNA is a contributor of 8-OHdG production associated with ROS (from NADH oxidase) during exercise (Radák et al., 1999). During exercise, mitophagy (lysosomal degradation of defective mitochondria) increases 8-OHdG production (MacDougall et al., 1979; Ruple et al., 2021; Schofield and Schafer, 2021). With higher mitochondria content in muscle, trained individuals are anticipated to exhibit a more pronounced rate of mitochondrial DNA turnover than untrained individuals. Furthermore, training adaptation can lower the demand for recruiting bone marrow stem cells into circulation against an acute bout of aerobic exercise (Ryan et al., 2018). Bone marrow stem cells lower the levels of ROS, evidenced by a decreased 8-OHdG in cells treated with exosomal molecules released from bone marrow stem cells (Damania et al., 2018). Bone marrow stem cells mobilize into circulation based on the magnitude of tissue damage (Ribeiro et al., 2017; Schmid et al., 2021) and inflammation (Agha et al., 2018). Therefore, the large effect of decreasing 8-OHdG levels after an acute bout of aerobic exercise among untrained individuals may be partly associated with greater levels of bone marrow cell mobilization compared to trained individuals.

The present quantitative analysis demonstrates an inverse correlation between exercise duration and average circulating 8-OHdG levels collected from 230 participants in 23 time points (*r* = −0.74). However, we need to be aware that intensity and duration are interrelated, as long-duration exercise cannot be sustained at high intensity. We found that post-exercise 8-OHdG levels increase above baseline in studies employing short-duration exercise (<30 min) but tend to decrease below the baseline level among studies employing long-duration exercise (>30 min). The linear relationship between exercise duration and circulating 8-OHdG levels is contributed by a mixed influence of the training status and exercise intensity. Studies on short-duration exercise mostly involved trained participants, whereas studies on long-duration exercise (>30 min) involved untrained participants. Similar to resistance exercise, high-intensity aerobic exercise (≥75% VO_2max_, duration from 5–45 min, four studies) consistently increases circulating 8-OHdG levels regardless of duration and training status (Sacheck et al., 2003; Bloomer et al., 2006; Harms-Ringdahl et al., 2012; Pittaluga et al., 2013).

The significant 8-OHdG lowering effect comes from only two studies (involving untrained participants) using moderate-intensity long-duration aerobic exercise (Sato et al., 2003; Itoh et al., 2006). It is likely that the DNA repair mechanism is upregulated gradually during prolonged exercise to lower circulating 8-OHdG levels originating from contracting muscle. This possibility is suggested by the evidence of increased excision of ^32^P-labeled damaged oligonucleotide in exercising muscle (Radák et al., 2002). Furthermore, exercise also increases DNA damage repair enzyme 8-oxoguanine-DNA glycosylase (OGG1) in skeletal muscle (Radak et al., 2009).

One limitation of this meta-analysis is the lack of percent contribution of bias of the 16 included studies. To obtain certainty of the evidence in greater detail, CINeMA software may be a future option for this type of meta-analysis. Furthermore, the studies included in this meta-analysis come from blood sample assessment. Only three studies reporting 8-OHdG levels in human skeletal muscle are currently available (Radák et al., 1999; Fogarty et al., 2013; Yasuda et al., 2015). Two studies have shown an increased 8-OHdG in human skeletal muscle after resistance exercise (Radák et al., 1999; Fogarty et al., 2013). However, aerobic cycling exercise for up to 5 h showed minimal changes in muscle 8-OHdG (Yasuda et al., 2015). The effects of exercise on 8-OHdG in human skeletal muscle and the role of 8-OHdG in muscle inflammation following a single bout of exercise require more investigation.

## Conclusion

8-OHdG has emerged as a molecular signal that gauges the levels of exercise-induced damage and stimulates a corresponding regeneration mechanism during inflammation. In this quantitative analysis, the results from pooled studies confirmed increased levels of circulating 8-OHdG after resistance exercise and high-intensity aerobic exercise. For aerobic exercise, this acute response varied depending on an individual’s training status. The fact that 8-OHdG treatment can accelerate the resolution of inflammation suggests that this molecule underlies training adaptation.

## Data Availability

The original contributions presented in the study are included in the article/Supplementary Material; further inquiries can be directed to the corresponding author.
