# Supplementary material for: DNA oxidation after exercise: a systematic review and meta-analysis
Source: Front Physiol. 2023 Oct 31;14:1275867. doi: 10.3389/fphys.2023.1275867 (PMC10644354; doi:10.3389/fphys.2023.1275867)
Supplement: Supplementary file 1 [file Table1.docx]

DNA oxidation after exercise: A systematic review and meta-analysis

Mengxin Ye ^1,2^, Luthfia Dewi ^2^, Yu-Chieh Liao ^2^, Andrew Nicholls ^2^,

Chih-Yang Huang ^4,5,6,7,8^, Chia-Hua Kuo^2,3*^

^1^ College of Physical Education and Science, Zhejiang Normal University, Jinhua 321004, China

^2^ Laboratory of Exercise Biochemistry, University of Taipei, Tianmu Campus, Taipei 11153, Taiwan

^3^ School of Physical Education and Sports Science, Soochow University, Suzhou, China.

^4^ Cardiovascular and Mitochondria Related Disease Research Center, Hualien Tzu Chi Hospital, Buddhist Tzu Chi Medical Foundation, Hualien, Taiwan

^5^ Department of Medical Research, China Medical University Hospital, China Medical University, Taichung, Taiwan

^6^ Department of Biotechnology, Asia University, Taichung, Taiwan

^7^ Center of General Education, Buddhist Tzu Chi Medical Foundation, Tzu Chi University of Science and Technology, Hualien, Taiwan

^8^ Graduate Institute of Basic Medical Science, China Medical University, Taichung City, Taiwan

Correspondence should be addressed to

Chia-Hua Kuo, Ph.D.

Laboratory of Exercise Biochemistry

University of Taipei, Taipei, Taiwan

E-mail: kuochiahua@gmail.com; kch@utaipei.edu.tw

Phone: +886-970540921; Fax: +886-2-28753383

ORCID ID: [https://orcid.org/0000-0002-1731-4984](https://orcid.org/0000-0002-1731-4984?lang=zh_TW)

**Supplementary Table S1** PICOS model used to perform the meta-analysis

| **Criteria** | **Included** | **Excluded** |
| --- | --- | --- |
| Population | Humans | Animals |
| Intervention | Acute, aerobic and resistance exercise | Training (chronic) and anaerobic exercise |
| Comparison | Pre-exercise baseline |  |
| Outcome | 8-OHdG | Comet assay |
| Study type | Randomized and non-randomized trials |  |
| 8-OHdG, 8-hydroxy-2′-deoxyguanosine. | | |
