# Supplementary material for: DNA oxidation after exercise: a systematic review and meta-analysis
Source: Front Physiol. 2023 Oct 31;14:1275867. doi: 10.3389/fphys.2023.1275867 (PMC10644354; doi:10.3389/fphys.2023.1275867)
Supplement: Supplementary file 2 [file Table2.docx]

**SUPPLEMENTAL TABLE 2.** Risk of bias included studies using criteria of a revised Cochrane risk-of-bias tool for randomized trials

| **Studies with protocol** | **D1a** | **D1b** | **D2** | **D3** | **D4** | **D5** | **Overall** |
| --- | --- | --- | --- | --- | --- | --- | --- |
| Arazi et al. 2019 [1] |  |  |  |  |  |  |  |
| Bloomer et al. 2005 [2] |  |  |  |  |  |  |  |
| Bloomer et al. 2007 [3] |  |  |  |  |  |  |  |
| Çakır-Atabek et al. 2015 [4] |  |  |  |  |  |  |  |
| Fogarty et al. 2013 [5] |  |  |  |  |  |  |  |
| Mohammadjafari et al. 2019 [6] |  |  |  |  |  |  |  |
| Ra et al. 2013 [7] |  |  |  |  |  |  |  |
| Sarmiento et al. 2016 [8] |  |  |  |  |  |  |  |
| Bloomer et al. 2006 [9] |  |  |  |  |  |  |  |
| Harms-Ringdahl et al. 2012 [10] |  |  |  |  |  |  |  |
| Itoh et al. 2006 [11] |  |  |  |  |  |  |  |
| Pittaluga et al.2013 [12] |  |  |  |  |  |  |  |
| Sacheck et al. 2003 [13] |  |  |  |  |  |  |  |
| Saritaş et al. 2011 [14] |  |  |  |  |  |  |  |
| Sato et al. 2003 [15] |  |  |  |  |  |  |  |
| Shi et al. 2007 [16] |  |  |  |  |  |  |  |

D1a: Randomization process

D1b: Timing of identification of recruitment

D2: Deviations from the intended interventions

D3: Missing outcome data

D4: Measurement of the outcome

D5: Selection of the reported result

Low risk

Some concerns

High risk
