# Supplementary material for: DNA oxidation after exercise: a systematic review and meta-analysis
Source: Front Physiol. 2023 Oct 31;14:1275867. doi: 10.3389/fphys.2023.1275867 (PMC10644354; doi:10.3389/fphys.2023.1275867)
Supplement: Supplementary file 3 [file Table3.docx]

**SUPPLEMENTAL TABLE 3.** Study characteristics of included studies

| **Study/year** | **Sample size/age** | **Training status** | **Exercise type**  **(intensity, duration)** | **Specimens** | **Follow-up time (h)** | **8-OHdG change from pre-exercise (%)** |
| --- | --- | --- | --- | --- | --- | --- |
| Arazi et al. 2019 [1] | Male (n = 8)  Aged 21 y | Trained | Resistance exercise  (jumps, 15 sets of 10 maximal-effort verticals) | Serum (ELISA) | 0  1 | +18  +36 |
| Bloomer et al. 2005 [2] | Male (n = 10)  Aged 24 y | Trained | Resistance exercise  (squat, 70% 1RM) | Serum (ELISA) | 24 | +18 |
|  |  |  | Aerobic exercise  (cycle, 70% V̇O_2max_, 30 min) |  | 24 | +1 |
| Bloomer et al. 2007 [3] | Male (n = 13)  Aged 24 y | Trained | Resistance exercise  (squat, 70% 1RM,1 set of 15 reps) | Plasma (ELISA) | 0 | +21 |
| Çakır-Atabek et al. 2015 [4] | Male (n = 8)  Aged 26 y | Untrained | Resistance exercise  (leg extension, 50% - 90% 1RM, 6 sets, 90-120 s of rest between sets) | Serum (ELISA) | 0  0.5  1  24 | +6  +19  +16  +15 |
|  | Male (n = 8)  Aged 29 y | Trained |  |  | 0  0.5  1  24 | +22  +4  -2  +7 |
| Fogarty et al. 2013 [5] | Male (n = 6)  Aged 27 y | Untrained | Resistance exercise  (knee extension, 100 reps) | Plasma (ELISA) | 0 | +40.4 |
| Mohammadjafari et al. 2019 [6] | Male (n = 15)  Aged 28 y | Trained | Resistance exercise  (5 sets, 80% 1RM, 6 actions) | Serum (ELISA) | 0 | +23.1 |
| Ra et al. 2013 [7] | Male (n = 9)  Aged 22 y | Untrained | Resistance exercise  (45° flexion angle, 90% MVC, 6 sets, 5 reps) | Serum (ELISA) | 48 | +1 |
| Sarmiento et al. 2016 [8] | Male (n = 34)  Aged 38.2 y | Trained | Resistance exercise  (Exercise protocol consisted of 10 resistance body-building strenuous exercises, 60 - 70% 1RM) | Plasma (ELISA) | 0  24 | +13  +11 |
| Bloomer et al. 2006 [9] | Male (n = 11)  Female (n = 6)  Aged 23 y | Trained | Aerobic exercise  (run, 80% V̇O_2max_, 30 min) | Serum (ELISA) | 0 | +5 |
| Harms-Ringdahl et al. 2012 [10] | Female (n = 7)  Aged 29 y  Male (n = 8)  Aged 32 y | Untrained | Aerobic exercise  (cycle,80% HR_max_ ≈ 70% VO_2max_, 20 min) | Serum (ELISA) | 1 | +42 |
| Itoh et al. 2006 [11] | Male (n = 8)  Aged 21 y | Untrained | Aerobic exercise  (run,75% HR_max_ ≈ 60% VO_2max_, 10 km ≈ 65 min) | Plasma (ELISA) | 0  1  24 | -40  -35  -29 |
| Pittaluga et al.2013 [12] | Female (n = 7)  Aged 68 y | Trained | Aerobic exercise  (cycle, incremental exercise test to exhaustion, 5.5 min) | Serum (ELISA) | 0.5  24 | +6  +23 |
| Sacheck et al. 2003 [13] | Male (n = 8)  Aged 26 y | Untrained | Aerobic exercise  (run, 75% VO_2max_, 45 min) | Leukocytes (HPLC) | 24 | +1 |
|  | Male (n = 8)  Aged 71 y |  |  |  | 24 | +11 |
| Saritaş et al. 2011 [14] | Male (n = 22)  Aged 22 y | Trained | Aerobic exercise  (run, maximal running distance for VO_2max_ measurement, 12 min) | Serum (ELISA) | 0  24 | +33  +52 |
| Sato et al. 2003 [15] | Male (n = 8)  Aged 22 y | Untrained | Aerobic exercise  (cycle, 50% VO_2max,_ 30 min) | Leukocyte DNA (HPLC) | 0  1  24  48 | -24  -20  -27  -32 |
|  | Male (n = 7)  Aged 21 y | Trained |  |  | 0  1  24  48 | -9  +6  +13  +10 |
| Shi et al. 2007 [16] | Male (n = 10)  Aged 22-38 y | Trained | Aerobic exercise  (cycle, 50% VO_2max_, 8.5-15.5 min) | Leukocytes (HPLC) | 0  3  24 | +34  +21  +34 |

*8-OHdG* 8-hydroxy-2′-deoxyguanosine, *ELISA* enzyme-linked immunosorbent assay, *HPLC* high-performance liquid chromatography, *RM* repetition maximum, *VO_2max_* maximal oxygen consumption, *HR_max_* heart rate max
